# LINC00586 Represses ASXL1 Expression Thus Inducing Epithelial-To-Mesenchymal Transition of Colorectal Cancer Cells Through LSD1-Mediated H3K4me2 Demethylation

**DOI:** 10.3389/fphar.2022.887822

**Published:** 2022-05-02

**Authors:** Fengting Liu, Xiaofang Ma, Xiyun Bian, Chunyan Zhang, Xiaozhi Liu, Qiang Liu

**Affiliations:** ^1^ Tianjin Key Laboratory of RadiationMedicine and Molecular Nuclear Medicine, Institute of Radiation Medicine, Chinese Academy of Medical Sciences and Peking Union Medical College, Tianjin, China; ^2^ Medical Research Center, The Fifth Central Hospital of Tianjin, Tianjin, China; ^3^ Tianjin Key Laboratory of Epigenetics for Organ Development in Preterm Infants, The Fifth Central Hospital of Tianjin, Tianjin, China

**Keywords:** colorectal cancer, epithelial-to-mesenchymal transition, LINC00586, LSD1, ASXL1

## Abstract

Colorectal cancer (CRC) is a major public health problem on a global scale by virtue of its relatively high incidence. The transition of tumor cells from an epithelial to a mesenchymal-like phenotype, so-called epithelial-to-mesenchymal transition (EMT), is a key hallmark of human cancer metastasis, including CRC. Understanding the signaling events that initiate this phenotypic switch may provide opportunities to limit the metastasis of CRC. In this study, we aim to identify long non-coding RNA (lncRNA) mediated epigenetic regulation under the context of CRC. 54 paired samples of tumor tissues and surrounding non-tumor tissues were collected from CRC patients. Cultured human CRC cells HCT116 and LoVo were assayed for their viability and migration using CCK-8 tests and transwell migration assays. The expression of EMT-specific markers (E-cadherin, N-cadherin and vimentin) was analyzed biochemically by RT-qPCR and immunoblot analyses. Interaction among LINC00586, LSD1, and ASXL1 was determined by RNA immunoprecipitation and chromatin immunoprecipitation. *In vivo* analysis of LINC00586 was performed in nude mice xenografted with HCT116 cells. LINC00586 was overexpressed in CRC tissues and associated with patient survival. LINC00586 knockdown repressed HCT116 and LoVo cell viability, migration, their phenotypic switch from epithelial to a mesenchymal, and tumorigenesis *in vivo*. We demonstrated LINC00586 recruited the LSD1 into the ASXL1 promoter region and epigenetically silenced the ASXL1 expression. An ASXL1 gene resisting to LINC00586 attack was demonstrated in cultured HCT116 and LoVo cells and mouse xenograft models of human CRC. Overall, discovery of the LINC00586/LSD1/ASXL1 axis partially explains epigenetic mechanism regulating EMT in CRC, providing a therapeutic target to limit CRC metastasis.

## Introduction

Colorectal cancer (CRC) is known as the common type of gastrointestinal tract cancers worldwide (Rabeneck et al., 2020). The American Cancer Society updates CRC occurrence that approximately 147,950 individuals will be diagnosed with CRC and 53,200 will die from the disease in 2020 (Siegel et al., 2020). About 90% of CRC-related deaths are attributed to distant metastasis (Ding et al., 2018). Routine clinical management of patients with metastatic colorectal cancer includes oxaliplatin-containing therapy followed by irinotecan-containing therapy at progression (Pfeiffer et al., 2020; Saleh et al., 2020). Although the advancement has been achieved for treating metastatic CRC patients, their overall survival remains unsatisfactory (Basile et al., 2020). A process of tumor cells switch from an epithelial to a mesenchymal-like phenotype is termed epithelial mesenchymal transition (EMT), which is a distinctive feature of human cancers including CRC (Mittal, 2018; Kim et al., 2020). Yet, it still needs to make great efforts to discover and improve therapeutic targets for some obstacles to effective therapy (Fan and Liu, 2017). It is an urgent need to develop the potential effective targets for CRC diagnosis and therapy, such as long non-coding RNAs (lncRNAs) that exert great effects on the progression of CRC.

LncRNAs play a crucial role in the regulation of tumorigenesis, and their aberrant expression underpins the progression of CRC (Galamb et al., 2020). LINC00586, also known as BRAF-activated lncRNA (BANCR), is a 693 bp lncRNA located on chromosome 9, being implicated in the development of CRC and demonstrated to promote the tumorigenesis in CRC (Ma et al., 2018). A previous study clarified that LINC00586 induces the migration of CRC cells and promotes the process of EMT (Guo et al., 2014). Of note, researchers performed quantitative proteomics strategy to globally identify LINC00586-regulated proteins in HeLa cells and found 569 differentially expressed proteins (DEPs) in HeLa cells upon knockout of BANCR (Wang et al., 2022). Nevertheless, the detailed molecular mechanism underlying LINC00586 waits to be further elucidated. LncRNAs as critical contributors in the epigenetic mechanisms are involved in the initiation, progression and metastasis of CRC (Ghafouri-Fard et al., 2021). Furthermore, lncRNAs function in many cases as transcriptional regulators by binding to histone-modifying complexes, to DNA binding proteins (including transcription factors), and even to RNA polymerase II (Long et al., 2017). Transcriptional Regulator 1 (ASXL1), based on the RNA-protein interaction prediction tool (http://pridb.gdcb.iastate.edu/RPISeq/). It has been previously addressed that depleted ASXL1 might be related to lymphatic invasion of CRC, which suggests that ASXL1 is likely to function as a tumor suppressor in CRC (Lee et al., 2020). A direct interaction of ASXL1 with histone H3 demethylase lysine-specific demethylase 1 (LSD1) through the N-terminal region nearby the HP1-binding site was previously reported (Lee et al., 2010). LSD1 could participate in the regulation of CRC by interacting with tetraspanin 8 (Zhang et al., 2020). LSD1 was found to downregulate CDH-1 expression by epigenetic modification and consequently facilitate the metastasis of colon cancer cells (Ding et al., 2013). Taken above into consideration, we aim at investigating the role of LINC00586 in progression of CRC by recruitment of LSD1 into the ASXL1 promoter region and epigenetically silencing the ASXL1 expression. For this purpose, we determine expression pattern of LINC00586 in CRC tissue sample and its association with CRC progression and prognosis. We also tested the effect of LINC00586 on CRC cells *in vitro* setting. To further validate results *in vivo*, we xenografted human CRC cells into nude mice to examine the effect of LINC00586 on the *in vivo* tumorigenesis of CRC cells.

## Materials and Methods

### Ethics Statement

Human tissue specimens were collected from CRC patients who signed informed consent for a protocol approved by the Ethics committee of the hospital. The experiments involved animals were performed with the approval from the institutional animal care and use committee of the hospital.

### Human Tissue Specimen Collection

Tumor tissues and matched surrounding non-tumor tissues were collected from 54 CRC patients who were admitted into our hospital between June 2017 and June 2018. The diagnosis of these CRC patients was confirmed by histopathological examinations and their clinical stages were the 7th edition of American Joint Committee on Cancer (AJCC) Cancer Staging Manual, TNM staging criteria for CRC (Edge and Compton, 2010). None received radiotherapy and chemotherapy before tissue specimen collection. Medical records were available from each patient. All patients were followed up 36 months by telephone or hospital visit until June 2021. The follow-up ranged from 2 to 36 months.

### Cell Harvest and Transient Transfection

Normal human colon epithelial cells (FHC) and human CRC cell lines (HCT116, LoVo, HT-29, SW480, and SW620) were purchased from the Cell bank in Shanghai Institutes of Chinese Academy of Sciences and harvested in the RPMI-1640 supplemented with 10% fetal bovine serum (FBS) and 1% antibiotics (penicillin/streptomycin). Cell harvest was performed in the incubator (37°C, 5 %CO_2_). siRNA against LINC00586 and pcDNA expression vector containing the full-length of human LINC00586 transcripts were purchased from GenePharma (Shanghai, China) and delivered into HCT116 and LoVo cells to specifically blunt and overexpress LINC00586 in CRC cells, respectively, using lipofectamine 3000 reagent (Invitrogen, United States) according to the manufacturer’s recommendation. Likewise, ASXL1 overexpression was achieved in HCT116 and LoVo cells using pcDNA expression vector containing the full-length of human ASXL1 gene (Shanghai GenePharma Co., Ltd., China).

### Real-Time Quantitative PCR (RT-qPCR) Analysis

Total RNA was extracted from tissues and cells using TRIzol reagents (Invitrogen). cDNA was generated using PrimeScript RT kit (RR036A, Takara) as per the manufacturer’s protocol. qRT-PCR was performed with the SYBR®Premix ExTaqTM II (RR820A, Takara) on the ABI PRISM®7300 System (Applied Biosystems). Data were normalized to fold change of GAPDH, a house-keeping gene, and relative expressions of mRNAs of interest were determined using the delta-delta comparative threshold cycle (ΔΔCt) methods. Primers are listed in Table 1.

**TABLE 1:** Primer sequences used for RT-qPCR.

Target	Primer sequences (5′-3′)
LINC00586	F: ACA​GGA​CTC​CAT​GGC​AAA​CG
	R: ATG​AAG​AAA​GCC​TGG​TGC​AGT
ASXL1	F: GGT​CCT​GTC​TCA​GTC​CCT​CA
	R: ATA​ACC​ACG​GGG​TCA​GAG​GT
E-cadherin (human)	F: AAG​GCA​CGC​CTG​TCG​AAG​CA
	R: ACG​TTG​TCC​CGG​GTG​TCA​TCC​T
N-cadherin (human)	F: TGC​GCG​TGA​AGG​TTT​GCC​AGT
	R: TGG​CGT​TCT​TTA​TCC​CGG​CGT
Vimentin (human)	F: ACC​GCA​CAC​AGC​AAG​GCG​AT
	R: CGA​TTG​AGG​GCT​CCT​AGC​GGT​T
E-cadherin (mouse)	F: TAC​CAT​GCT​GGT​AGG​GTG​GA
	R: GTG​GGA​GTC​AAA​TCC​CGG​TT
N-cadherin (mouse)	F: ACC​ACT​GGC​AAG​TTC​ACA​GC
	R: TGT​ACC​CCA​ACT​AGT​CGC​CA
Vimentin (mouse)	F: TCC​ACC​CCT​AGC​CTG​ATA​CC
	R: TGC​TGA​CTT​AAA​GGG​GAC​CA
GAPDH	F: AAC​GGA​TTT​GGT​CGT​ATT​GGG
	R: TCG​CTC​CTG​GAA​GAT​GGT​GAT

F, forward; R, reverse.

### Subcellular Fractionation

HCT116 and LoVo cells were harvested for subcellular RNA isolation. Cell cytoplasm and nuclear fractionation was performed with SurePrep™ Nuclear or Cytoplasmic RNA Purification Kit (Fisher Scientific, MA) in accordance with provider’s manual. RNA quality and quantity were determined with Nanodrop 2000 (Invitrogen, MA). The U6 and GAPDH transcripts were employed as nuclear and cytosolic controls, respectively.

### Immunoblotting

The whole-cell protein was extracted from cell lysates in protease inhibitors-contained radioimmunoprecipitation buffer. After SDS-PAGE separation and membrane transfer, the protein was probed with the following primary antibodies (Abcam, United Kingdom): Anti-ASXL1 antibody (ab228009), anti-SEMA6B antibody (ab180215), anti-E-cadherin antibody (ab15148), anti-N-cadherin antibody (ab18203), anti-vimentin antibody (ab137321), and anti-GAPDH antibody (ab9485). Immunoblots were exposed to horseradish peroxidase-coupled goat anti-rabbit immunoglobulin and enhanced chemiluminescence detection reagents (EMD Millipore, United States). Gray value of target protein bands was quantified using ImageJ software, with GAPDH used for normalization.

### Cell Viability Assays

A commercially available CCK-8 kit was used to examine the viability of HCT116 and LoVo cells. In brief, HCT116 and LoVo cells were independently cultured for 0, 24, 48, and 72 h, with 10 μL CCK-8 solution added at the end of each culture for an additional incubation 2 h. Absorbance (at 450 nm) was determined using the Microplate reader, with growth curves depicted.

### Cell Migration Assays

Transwell chamber systems of 24-well plate with an 8-μm pore were performed for cell migration assays. In brief, HCT116 and LoVo cells were adjusted using the serum-free DMEM (100 μL) and 200 μL of cell suspension were added into the upper chambers. Following 24-hour incubation at 37°C, the cells that transferred to Matrigel-coated lower chamber containing 10% FBS-supplemented DMEM (Invitrogen) were subject to 4% paraformaldehyde fixation and 0.05% crystal violet staining. Stained cells were counted in six random fields per well using the inverted microscope.

### RNA Immunoprecipitation

LINC00586 binding with LSD1 was evaluated using the Magna RIP™RNA-Binding Protein Immunoprecipitation Kit (Millipore, United States) following the manufacturer’s recommendation. In brief, two parts of cell extracts were incubated with either anti-LSD1 antibody (ab17721, Abcam) or nonspecific rabbit IgG (ab2410, Abcam) and immunoprecipitated with magnetic Protein A/G beads. Following proteinase k incubation, the immunoprecipitated RNA and total RNA from the whole cell lysates (input controls) were extracted for RT-qPCR analysis.

### Chromatin Immunoprecipitation-qPCR

Enrichment of LSD1 and H3K27me3 in the ASXL1 promoter region was evaluated by the EZ ChIP™Chromatin Immunoprecipitation Kit (Millipore) following the manufacturer’s recommendation. In brief, HCT116 and LoVo cells were fixed with 10% formaldehyde for 10 min to generate DNA-protein cross-links. Cell lysates were then sonicated to generate chromatin fragments. The chromatin fragments were incubated with Protiein G Agarose (1 h) and then centrifuged (5,000 g, 1 min). Cell extracts were split into four parts of which one was used as “Input” and others were probed with anti-LSD1 antibody (ab17721, Abcam), anti-H3K27me3 antibody (ab6002) or nonspecific rabbit IgG (ab2410, Abcam) at 4°C overnight. The DNA-protein complexes were precipitated by using protein G Agarose. The immunoprecipitation was de-crosslinked, and the DNA samples were extracted for real-time qPCR analysis.

### Constructs, Lentivirus Production, and Transduction

The recombinant lentivirus harboring shRNA targeting LINC00586 (5′-GGA​GTG​GCG​ACT​ATA​GCA​AAC-3′), scramble shRNA (5′-TTC​TCC​GAA​CGT​GTC​ACG​T-3′), the full length of human ASXL1 gene (NCBI reference sequence ID, NM_015338.6), or the full length of human LINC00586 transcripts (NCBI reference sequence ID, NR_047671.2) were constructed and purchased from Shanghai GeneChem Co., Ltd. (shanghai, China). The recombinant lentivirus (LV-sh-LINC00586, LV-ASXL1, and LV-LINC00586) were produced in 293T cells. Supernatants containing lentiviruses were harvested 48 h later. We performed subsequent purification using ultracentrifugation. HCT116 cells were transduced with the recombinant lentivirus plus 5 μg/ml of Polybrene (Huang et al., 2018). The infected HCT116 cells were selected by puromycin (1 μg/ml), and the puromycin-resistant colonies were subsequently selected, expanded, and analyzed.

### Mouse Xenograft Models of CRC

A total of 25 BALB/c mice aged 6–8 weeks and weighing 18–25 g (Hunan SJA Laboratory Animal Co., Ltd., China) were kept under specific pathogen-free conditions. HCT116 cells transduced with the recombinant lentivirus (2 × 10^6^ cells for each cell line) were resuspended in 200 ml phosphate buffered saline and subcutaneously injected into BALB/c mice. 2 weeks after implantation, the mice were euthanized, with CRC xenografts collected. The mice were euthanized by exposure to prolonged inhalatory anesthesia. Tremendous efforts were done to minimize pain the included animals suffered.

### Statistical Analysis

Measurement data were presented as mean ± standard deviation of three independent experiments (each in triplicate) and analyzed by paired or unpaired *t*-test, a one-way analysis of variance (ANOVA) with Tukey’s test, and repeated measurements ANOVA with Bonferroni corrections as required. Percentage or frequency was used to report enumeration data and chi-square test was used for statistical comparisons. Survival curves were plotted using Kaplan-Meier’s method, and statistical differences were identified by a log-rank test. Cox’s proportional hazards model was employed to identify the independent factors. Statistical comparisons were processed by SPSS 21.0 software (IBM, Armonk, NY, United States), with two-tailed *p* < 0.05 as a level of statistical significance.

## Results

### High Expression of LINC00586 was Associated With CRC Progression and Unfavorable Prognosis

**FIGURE 1:**
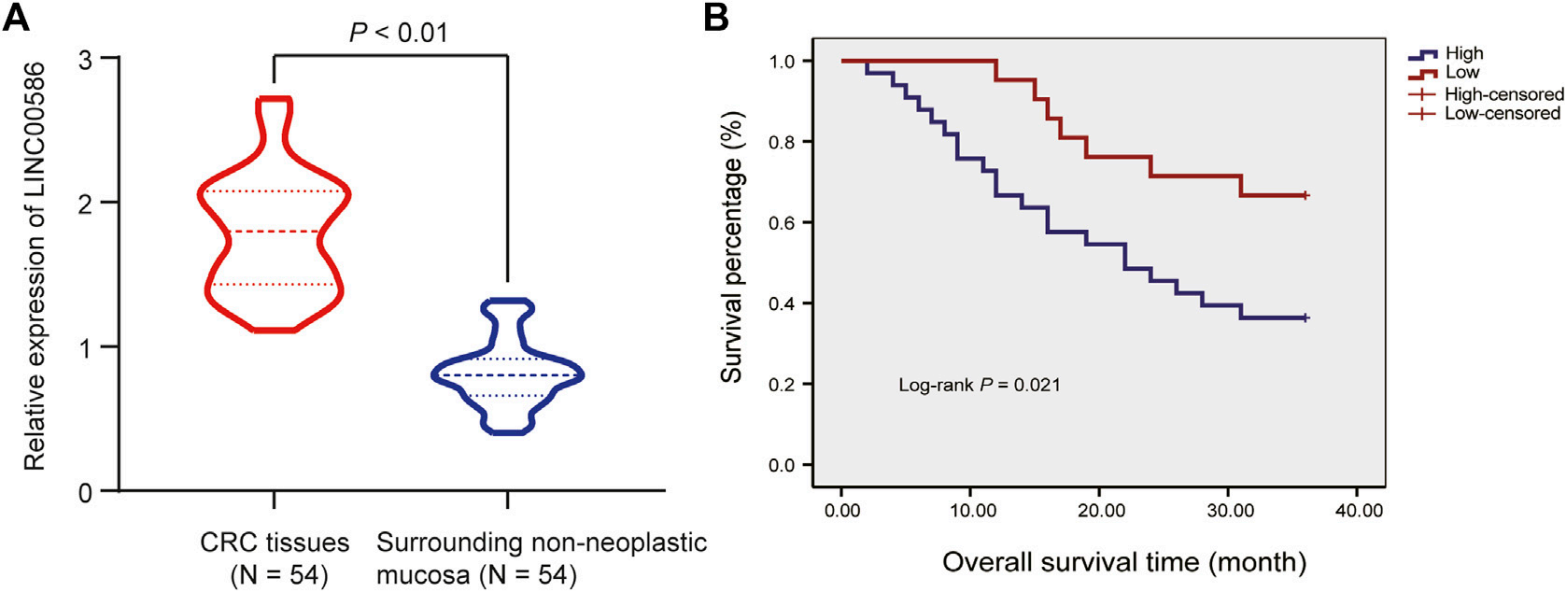
High expression of LINC00586 is associated with CRC progression and unfavorable prognosis. **(A)** The expression of LINC00586 was analyzed by real-time qPCR in CRC tissues (*n* = 54) and surrounding non-neoplastic mucosa (*n* = 54). **(B)** Overall survival and disease-free survival probabilities of 54 CRC patients were estimated according to LINC00586 expression using the Kaplan-Meier method and analyzed by Log-rank test. *p < 0.05 by paired *t* test.

We first set out to quantify the expression of LINC00586 in the clinical patient tissue samples. The real-time qPCR revealed an elevated LINC00586 in CRC tissues compared with surrounding non-neoplastic mucosa (Figure 1A). The expression of LINC00586 was associated with tumor node metastasis (TNM) and lymph node metastasis (LNM) of CRC patients (*p* < 0.05, Table 2). The median expression level of LINC00586 in tumor tissues was used to classify 54 CRC patients with high or low expression of LINC00586. The Kaplan-Meier curve was depicted to show CRC patient prognosis according to LINC00586 patients. Patients with high LINC00586 expression had shorter overall survival than those with low LINC00586 expression (*p <* 0.05, Figure 1B). Cox regression analysis revealed high LINC00586 expression, TNM, and LNM were independent prognostic factors for CRC (*p <* 0.05, Table 3). These data suggested that high expression of LINC00586 was associated with CRC progression and unfavorable prognosis.

**TABLE 2:** Correlation of LINC00586 expression with the clinical characteristics of CRC patients.

Clinical characteristics	Case	LINC00586 expression		Statistics	*p* value
		High (*n* = 33)	Low (*n* = 21)		
Age	54	50.78 ± 12.19	50.12 ± 13.29	*t* = 0.186	0.853
Gender	—	—	—	*χ* ^2^ = 0.691	0.406
Male	39	22	17	—	—
Female	15	11	4	—	—
Tumor size	—	—	—	*χ* ^2^ = 0.362	0.547
<4 cm	14	10	4	—	—
≥4 cm	40	23	17	—	—
Lymph node metastasis	—	—	—	*χ* ^2^ = 5.479	0.019*
No	24	10	14	—	—
Yes	30	23	7	—	—
Tumor differentiation	—	—	—	*χ* ^2^ = 0.084	0.772
High and medium	41	25	16	—	—
Low and none	13	8	5	—	—
TNM stage	—	—	—	*χ* ^2^ = 7.154	0.007*
Ⅰ-Ⅱ	25	10	15	—	—
Ⅲ-Ⅳ	29	23	6	—	—

Data were analyzed using *t* test or chi-square test (*p < 0.05).

**TABLE 3:** Cox regression analysis of independent prognostic factors for CRC.

	B	SE	Wald	df	Sig.	Exp(B)	95.0% CI	
							Upper	Lower
LINC00586	−1.610	0.813	3.919	1	0.048	0.200	0.041	0.984
LNM	−2.381	1.159	4.221	1	0.040	0.092	0.010	0.896
TNM	−1.634	0.817	3.998	1	0.046	0.195	0.039	0.968

### LINC00586 Knockdown Suppressed CRC Cell Viability, Invasion, EMT, and Tumorigenicity

**FIGURE 2:**
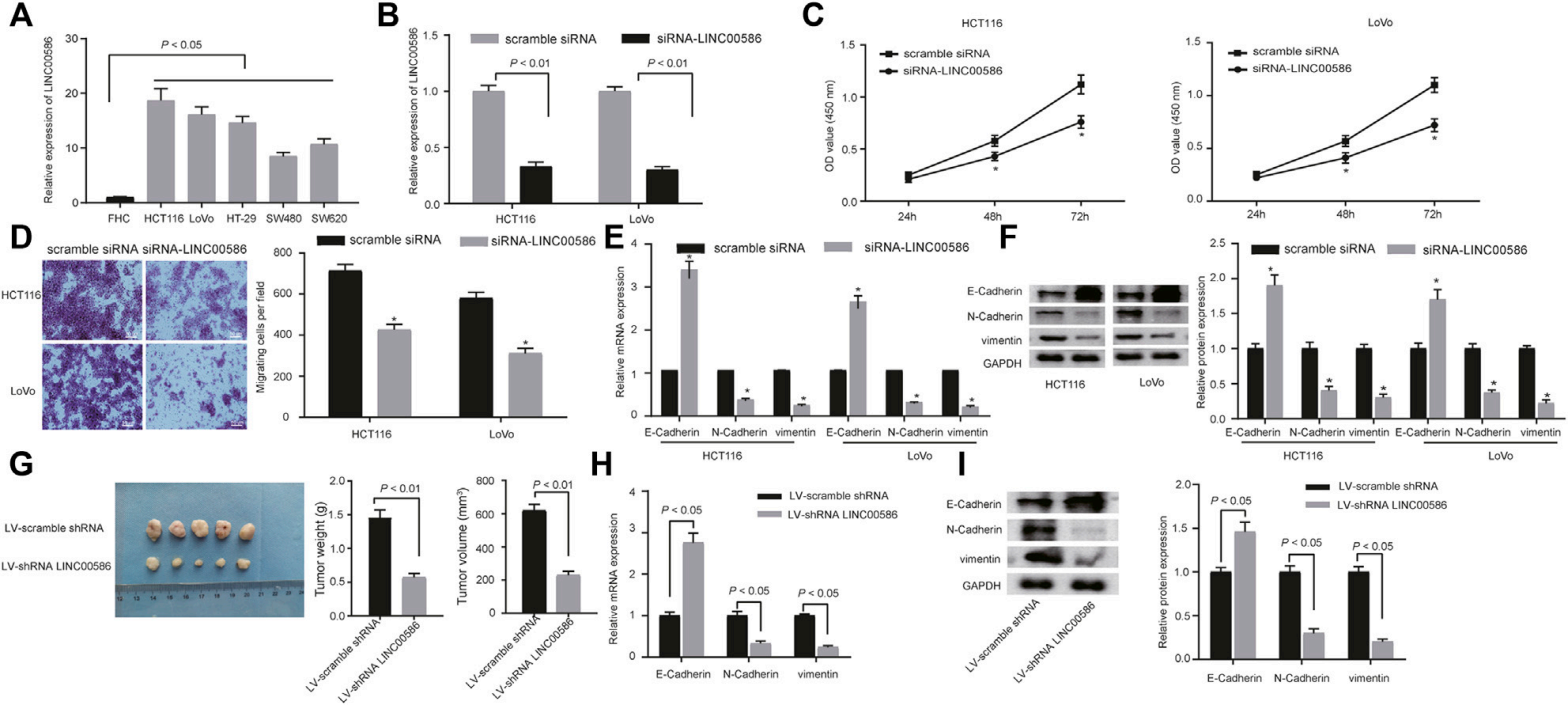
LINC00586 knockdown suppresses CRC cell viability, invasion, EMT, and tumorigenicity. **(A)** The expression of LINC00586 was analyzed by real-time qPCR in a panel of CRC cell lines (HCTl16, LoVo, HT-29, SW480, and SW620) and FHC. **(B)** Verification of LINC00586 knockdown in HCTl16 and LoVo cells by real-time qPCR. **(C)** The viability of HCTl16 and LoVo cells was examined by CCK-8 test upon LINC00586 knockdown. **(D)** Representative view (× 200) of HCTl16 and LoVo cells migrating from upper transwell chambers into lower ones and statistics of migrating cells upon LINC00586 knockdown. **(E)** The mRNA expression of E-Cadherin, N-Cadherin, and vimentin by real-time qPCR in HCTl16 and LoVo cells upon LINC00586 knockdown. **(F)** Immunoblots of E-Cadherin, N-Cadherin, vimentin, and their quantitative analysis in HCTl16 and LoVo cells upon LINC00586 knockdown. **(G)** Effects of LINC00586 knockdown on tumor growth in a xenograft mouse model. HCTl16 cells were infected with the recombinant lentivirus harboring shRNA targeting LINC00586 or scramble shRNA and then injected into the nude mice (*n* = 5), and the tumors were obtained 2 weeks after xenograft implantation and weighed. **(H)** The mRNA expression of E-Cadherin, N-Cadherin, and vimentin by real-time qPCR in CRC xenograft tissue sections upon LINC00586 knockdown. **(I)** Immunoblots of E-Cadherin, N-Cadherin, vimentin, and their quantitative analysis in CRC xenograft tissue sections upon LINC00586 knockdown. *p < 0.05 by unpaired *t* test or by repeated measurements ANOVA with Bonferroni corrections [only for **(C)**].

We next analyzed the expression of LINC00586 in a panel of CRC cell lines (HCT116, LoVo, HT-29, SW480, and SW620) and FHC by real-time qPCR. We observed a significant elevation of LINC00586 in all CRC cell lines when comparable to FHC (Figure 2A). HCT116 cells followed by LoVo possessed greatest fold change in LINC00586 expression. To experimentally examine the contribution of LINC00586 to CRC development, we constructed LINC00586-deficient stable CRC cells by delivering siRNA targeting LINC00586 into HCT116 and LoVo cells (Figure 2B). The cell viability evaluated by CCK-8 test was decreased by LINC00586 knockdown in HCT116 and LoVo cells (Figure 2C). Consistently, our transwell migration assays demonstrated HCT116 and LoVo cell migration was reduced upon LINC00586 knockdown (Figure 2D). We next characterized the functional role of LINC00586 in the process of EMT by determining the EMT markers E-Cadherin, N-Cadherin, and vimentin. The results of real-time PCR and immunoblotting analysis showed that LINC00586 knockdown resulted in an elevated E-Cadherin concomitant with declined N-Cadherin and vimentin in HCT116 and LoVo cells (Figures 2E,F). To further consolidate our preliminary observations *in vivo*, we xenografted LINC00586-deficient HCT116 cells into nude mice to examine the effect of LINC00586 on the *in vivo* tumorigenesis of HCT116 cells. As expected, lentivirus-mediated LINC00586 knockdown led to smaller tumors and displayed less weight and volume than corresponding control in mouse xenograft models (Figure 2G). Real-time PCR and immunoblotting analysis likewise revealed an elevated E-Cadherin concomitant with declined N-Cadherin and vimentin in CRC xenograft tissue sections upon LINC00586 knockdown (Figures 2H,I). Overall, these results support the notion that LINC00586 knockdown suppressed CRC cell viability, invasion, EMT, and tumorigenicity.

### High Expression of LINC00586 Underpins ASXL1 Inhibition in CRC Cells

**FIGURE 3:**
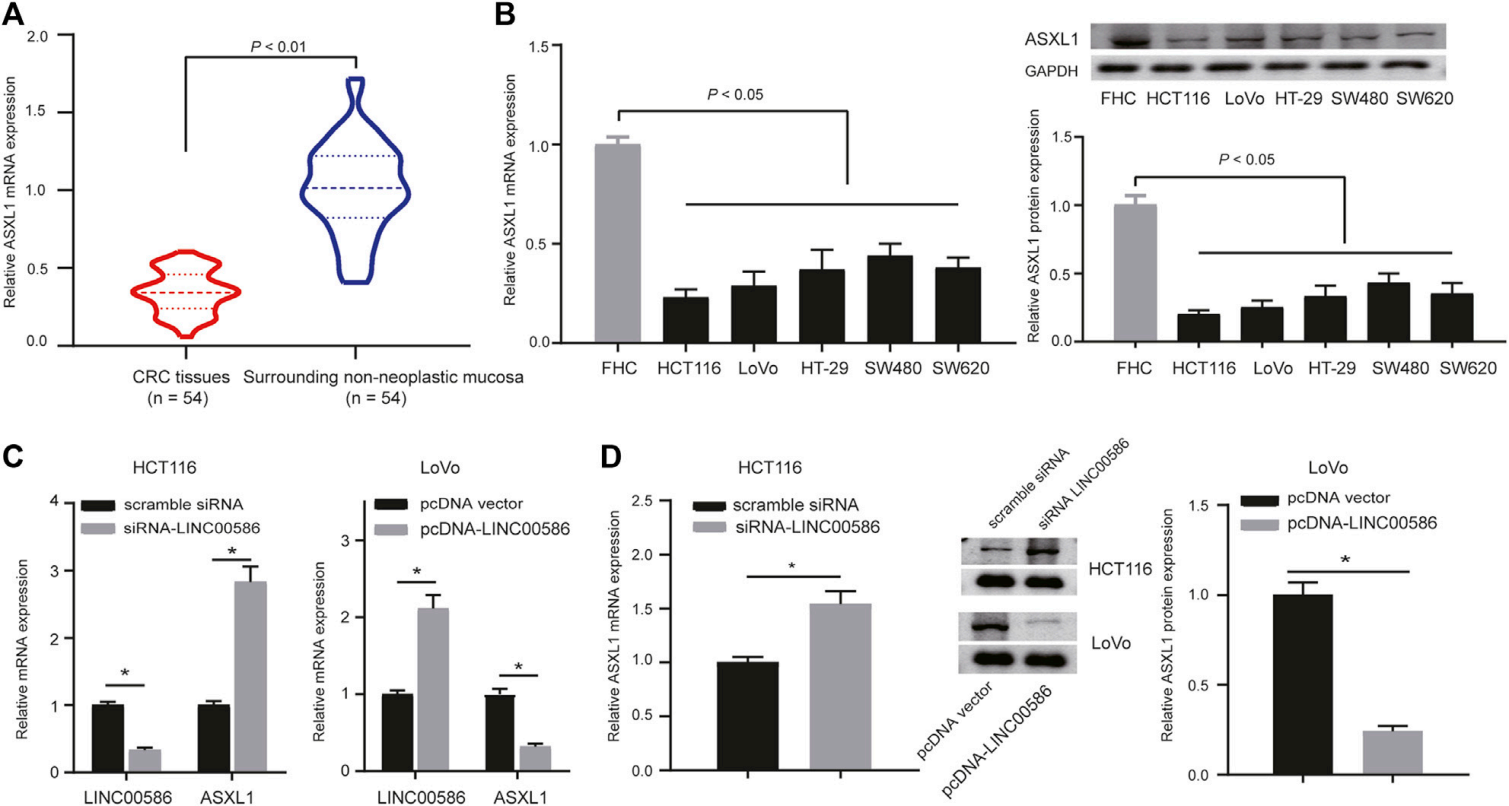
High expression of LINC00586 inhibits the expression of ASXL1 in CRC cells. **(A)** The mRNA and protein expression of ASXL1 was analyzed by real-time qPCR and immunoblotting analysis in CRC tissues (*n* = 54) and surrounding non-neoplastic mucosa (*n* = 54). **(B)** The mRNA and protein expression of ASXL1 was analyzed by real-time qPCR and immunoblotting analysis in a panel of CRC cell lines (HCTl16, LoVo, HT-29, SW480, and SW620) and FHC. **(C,D)**, The mRNA and protein expression of ASXL1 was analyzed by real-time qPCR **(C)** and immunoblotting analysis **(D)** in HCTl16 and LoVo cells upon LINC00586 knockdown or overexpression. *p < 0.05 by unpaired *t* test or by one-way ANOVA with Tukey’s test [only for **(B)**].

Subsequently, we are interested in the mechanism behind the regulation of LINC00586 in CRC. A computer-based RNA-protein interaction prediction (http://pridb.gdcb.iastate.edu/RPISeq/) revealed the binding relationship between LINC00586 and ASXL1. We next analyzed the expression of ASXL1 in the clinical patient samples and CRC cell lines. The real-time qPCR and immunoblotting analysis revealed lower expression levels of ASXL1 in CRC tissues (Figure 3A) and all CRC cell lines (Figure 3B) than surrounding non-neoplastic mucosa and FHC. To investigate whether ASXL1 could be regulated by LINC00586 in CRC cells, we determined mRNA and protein expression of ASXL1 in HCT116 and LoVo cells upon LINC00586 overexpression and knockdown by real-time qPCR and immunoblotting analysis. The results showed that LINC00586 knockdown upregulated ASXL1 expression, whereas ectopic expression of LINC00586 downregulated ASXL1 expression in HCT116 and LoVo cells (Figures 3C,D). Taken together, high expression of LINC00586 may underpins ASXL1 inhibition in the setting of CRC.

### LINC00586 Epigenetically Silenced ASXL1 Transcription Through LSD1-Mediated H3K4me2 Demethylation

**FIGURE 4:**
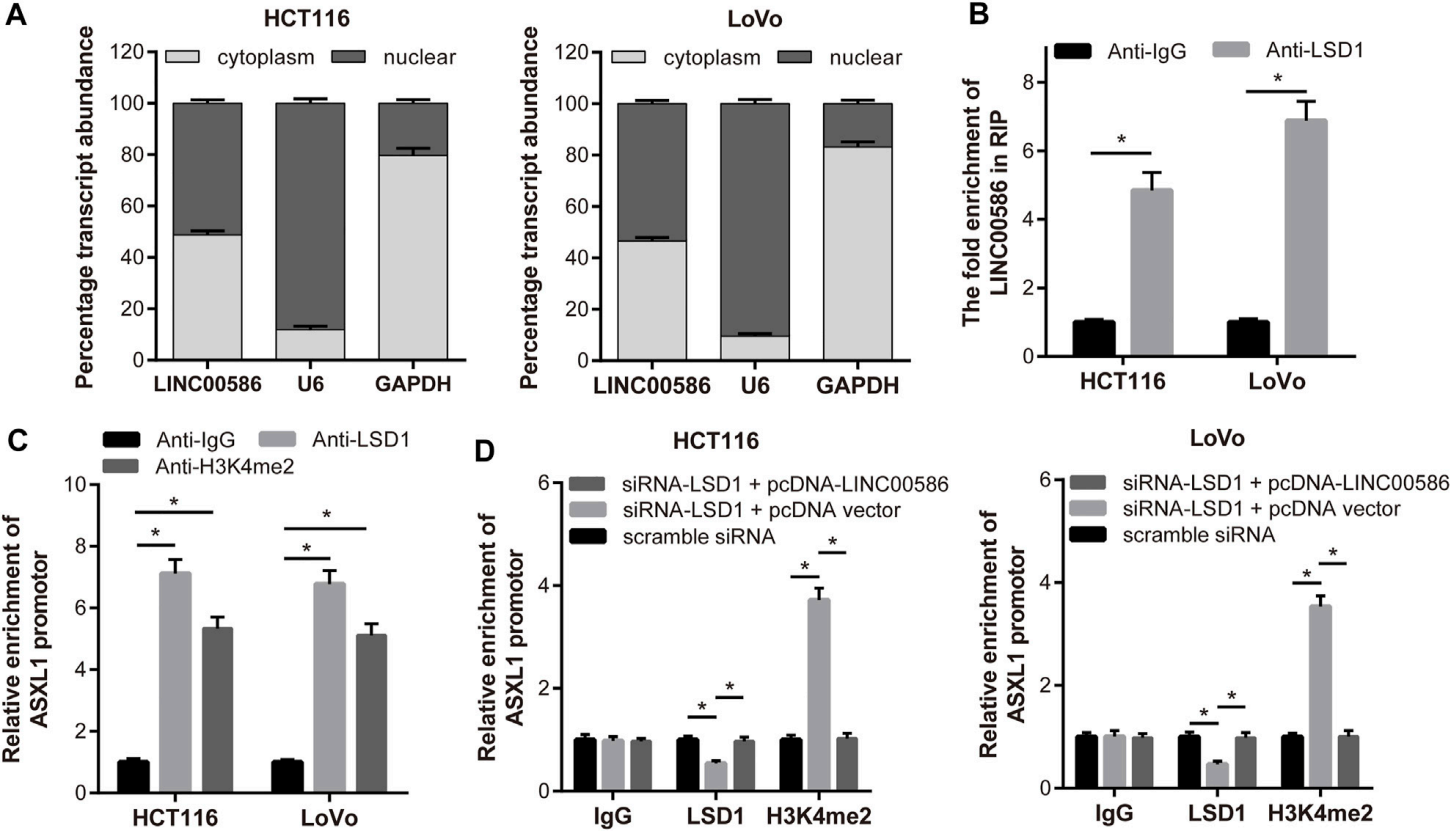
LINC00586 epigenetically silenced ASXL1 transcription through LSD1-mediated H3K4me2 demethylation. **(A)** Relative LINC00586 expression levels in nuclear and cytosolic fractions of HCTl16 and LoVo cells were determined by real-time qPCR. Nuclear controls: U6; Cytosolic controls: GAPDH. **(B)** RIP assay was performed to determine LINC00586 binding with LSD1. A significant enrichment of endogenous LINC00586 was detected in the anti-LSD1 RIP fraction (relative to nonspecific IgG control) in HCTl16 and LoVo cells. **(C)** HCTl16 and LoVo cells were subject to ChIP-PCR assays using anti-LSD1, anti-H3K4me2 or nonspecific IgG control for ASXL1 enrichment. **(D)** ChIP-PCR assays of ASXL1 enrichment in HCTl16 and LoVo cells following LSD1 knockdown and/or LINC00586 overexpression. *p < 0.05 by unpaired *t* test or by one-way ANOVA with Tukey’s test.

In this part, we set out to recapitulate the molecular mechanism by which LINC00586 regulated ASXL1 transcription. Considering that mechanisms of lncRNAs are largely dependent of specific cell locations, we first analyzed the subcellular localization of LINC00586 in both HCT116 and LoVo cells. As depicted in Figure 4A, we detected abundant LINC00586 transcripts in both cytoplasm and nuclear fractions, suggesting that LINC00586 may exert transcriptional regulation function on ASXL1. Given the critical role of LSD1 in epigenetic regulation of multiple target genes by lncRNAs, we performed RIP-qPCR assays and detected significant enrichment of endogenous LINC00586 in the anti-LSD1 RIP fraction (Figure 4B). Accordingly, LSD1 binding with the promoter region of ASXL1 was tested by ChIP assays using anti-LSD1, anti-H3K4me2, or nonspecific IgG control. We found a significant enrichment of ASXL1 in immunoprecipitated complex of anti-LSD1 antibody and anti-H3K4me2 antibody (Figure 4C), whereas we detected a reduced ASXL1 enrichment in anti-LSD1 immunoprecipitations but an increased enrichment in anti-H3K4me2 immunoprecipitations in HCT116 and LoVo cells transfected with siRNA targeting LSD1 compared with scramble siRNA (Figure 4D). Additionally, the ASXL1 enrichment in anti-LSD1 immunoprecipitations and anti-H3K4me2 immunoprecipitations upon LSD1 knockdown was greatly compromised by subsequent LINC00586 overexpression. All these data showed that LINC00586 epigenetically silenced ASXL1 transcription through LSD1-mediated H3K4me2 demethylation.

### LINC00586 Modulated CRC Cell Viability, Invasion, EMT, and Tumorigenicity by Inhibiting ASXL1

**FIGURE 5:**
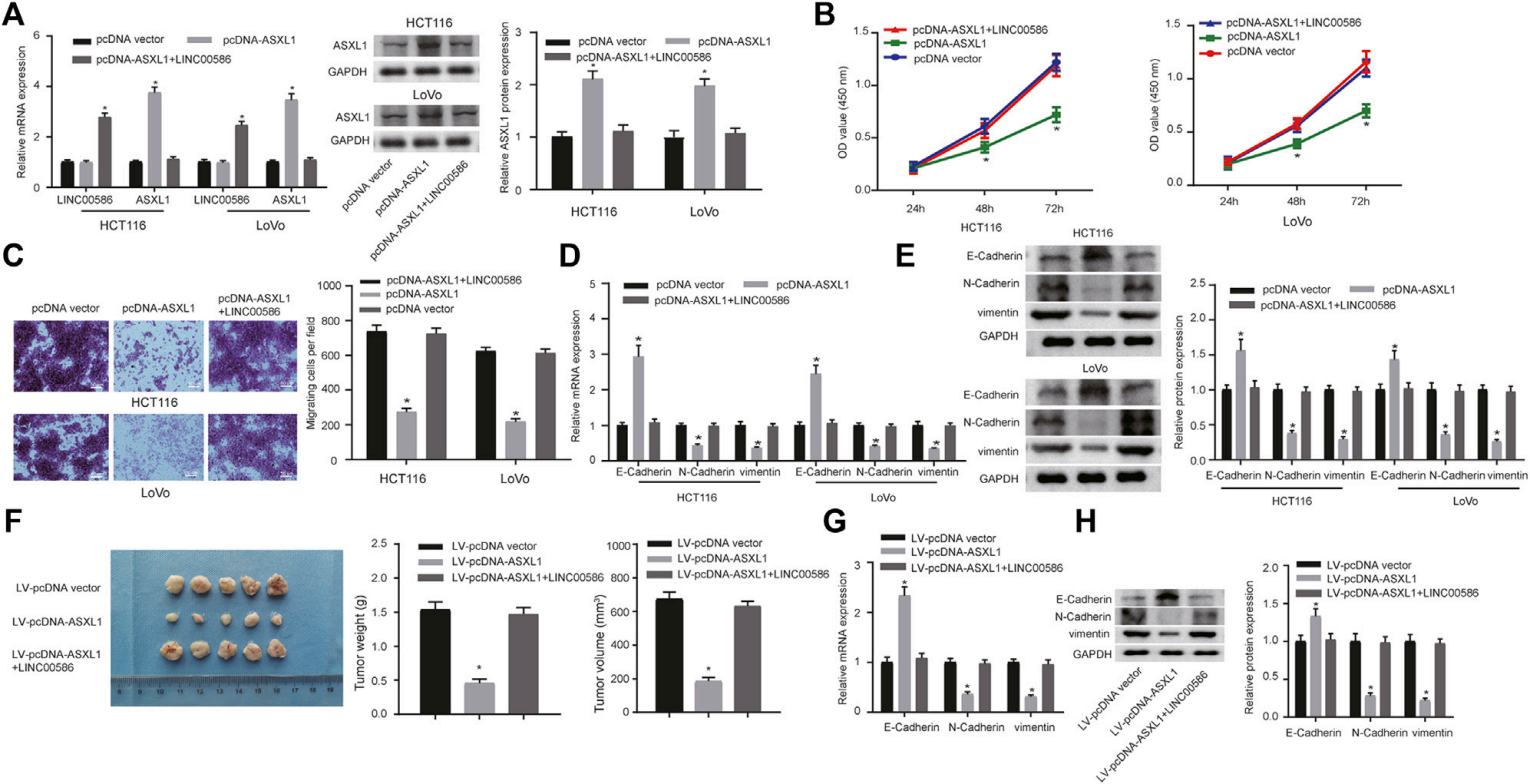
LINC00586 modulates CRC cell viability, invasion, EMT, and tumorigenicity by inhibiting ASXL1. **(A)** The mRNA and protein expression of ASXL1 was determined in HCTl16 and LoVo cells upon ASXL1 overexpression and/or LINC00586 overexpression by real-time qPCR and immunoblotting analysis. **(B)** The viability of HCTl16 and LoVo cells was examined by CCK-8 test upon ASXL1 overexpression and/or LINC00586 overexpression. **(C)** Representative view (× 200) of HCTl16 and LoVo cells migrating from upper transwell chambers into lower ones and statistics of migrating cells upon ASXL1 overexpression and/or LINC00586 overexpression. **(D)** The mRNA expression of E-Cadherin, N-Cadherin, and vimentin by real-time qPCR in HCTl16 and LoVo cells upon ASXL1 overexpression and/or LINC00586 overexpression. **(E)** Immunoblots of E-Cadherin, N-Cadherin, vimentin, and their quantitative analysis in HCTl16 and LoVo cells upon ASXL1 overexpression and/or LINC00586 overexpression. **(F)** HCTl16 cells were infected with the recombinant lentivirus harboring the full length of human ASXL1 gene alone or in combination with the recombinant lentivirus harboring human LINC00586 transcripts and injected into the nude mice (*n* = 5), and the tumors were obtained 2 weeks after xenograft implantation and weighed. **(G)** The mRNA expression of E-Cadherin, N-Cadherin, and vimentin by real-time qPCR in CRC xenograft tissue sections upon ASXL1 overexpression and/or LINC00586 overexpression. **(H)** Immunoblots of E-Cadherin, N-Cadherin, vimentin, and their quantitative analysis in CRC xenograft tissue sections upon ASXL1 overexpression and/or LINC00586 overexpression.

Since the observations of LINC00586 epigenetically silencing ASXL1 transcription, we aim at dissecting out whether LINC00586 is implicated in the development of CRC by inhibiting ASXL1. For this purpose, we first delivered pcDNA-ASXL1 into CRC cells to achieve ASXL1 overexpression in HCT116 and LoVo cells (Figure 5A). HCT116 and LoVo cells with ASXL1 overexpression were assayed to evaluate their viability and migration *in vitro*. The CCK-8 test and transwell migration assays revealed that ASXL1 overexpression suppressed CRC cell viability and migration (Figures 5B,C). Real-time qPCR and immunoblotting analysis results revealed an elevated E-Cadherin along with declined N-Cadherin and vimentin in HCT116 and LoVo cells upon ASXL1 overexpression (Figures 5D,E). More importantly, we observed ASXL1 overexpression coincident with LINC00586 overexpression not only significantly decreased the expression of ASXL1 but also compromised the inhibitory effects of ASXL1 on HCT116 and LoVo cell viability, migration, and the process of EMT. The *in vivo* analysis demonstrated ASXL1 overexpression attenuated the growth of subcutaneous xenotransplanted tumors of HCT116 cells (Figure 5F). Real-time qPCR (Figure 5G) and immunoblotting analysis (Figure 5H) likewise revealed an elevated E-Cadherin concomitant with declined N-Cadherin and vimentin in CRC xenograft tissue sections upon ASXL1 overexpression. We next established mouse xenograft models of HCT116 cells transfected with pcDNA-ASXL1+LINC00586 expression vector. Results revealed ensuing LINC00586 overexpression abrogated the tumor suppressive effects of ASXL1 on mouse xenograft models. Furthermore, we found the effects of ASXL1 on the expression of EMT markers were partially lost by subsequent LINC00586 overexpression mouse xenograft models. The aforementioned results unveiled that LINC00586 promoted CRC cell viability, invasion, EMT, and tumorigenicity by inhibiting ASXL1.

## Discussion

Increasing importance of lncRNAs, over 200 nucleotides in length, has been attached to the pathology of CRC, functioning as diagnostic and therapeutic biomarkers (Kim and Croce, 2018; Sun et al., 2018). LncRNAs are controllers of gene expression as well as a regulator of cell development, which includes cell migration, proliferation and apoptosis in carcinoma (Liz and Esteller, 2016). The aim of the current study was to explore the functional role of LINC00586 and the underlying mechanism in modulation of CRC cells to affect the development and progression of CRC. Collectively, the experimental data demonstrated that the LINC00586 promoted CRC cell viability, invasion, EMT, and tumorigenicity in association with ASXL1.

A fundamental finding of our study was to determine the expression profile of LINC00586. Previous evidence has manifested that lncRNAs are essential regulators of oncogenesis in CRC, which suggests that lncRNAs can function as biomarkers for CRC diagnosis (Silva-Fisher et al., 2020; Wu et al., 2020). It has been previously revealed that LINC00586 (BANCR) was aberrantly in various cancers (Zou et al., 2017). For instance, prior work reports that LINC00586 is highly expressed in melanoma cell lines and tissues, while knockdown of LINC00586 inhibits proliferation of melanoma cells (Li et al., 2014). Consistent with our study, it has been pointed out that LINC00586 is also highly expressed in CRC tissues and cell lines, the overexpression of which could promote cell migration by boosting EMT in ERK-dependent manner (Guo et al., 2014). Furthermore, LINC00586 has been identified to be associated with lymph node metastasis and poor survival of CRC patients (Shen et al., 2017). Likewise, we further found that knockdown of LINC00586 was able to suppress viability, invasion and EMT of CRC cells. All of these findings made our statement that LINC00586 played a pathogenic role in CRC reasonable.

Subsequently, mechanistic investigation showed that LINC00586 knockdown upregulated ASXL1 expression, whereas ectopic expression of LINC00586 downregulated ASXL1 expression in CRC cells. Aberrantly expressed ASXL1 has also been identified in CRC previously (Lee et al., 2019). However, we were the first reporting the regulatory relationship between LINC00586 and ASXL1. Moreover, ASXL1 can regulate H3K4me2 and H3K27me3 (Abdel-Wahab and Dey, 2013), which partially supports our proposal that LINC00586 epigenetically silenced ASXL1 transcription through LSD1-mediated H3K4me2 demethylation. LSD1 knockdown is reported to decline the proliferative rate of CRC cells (Ding et al., 2021). However, previous work also shows that low LSD1 expression and high H3K9me3 and H3K27me3 expression are both associated with more advanced stage of CRC (Carvalho et al., 2018). Therefore, how LSD1 exerted its function in the regulation of LINC00586/ASXL1 remains to be further investigated in the future study. Additionally, the data derived from our study showed that overexpression of ASXL1 was linked to the therapeutic role in CRC, which was manifested by elevated E-Cadherin and declined N-Cadherin and Vimentin levels. ASXL1 has already been identified as a tumor suppressor in CRC, which plays a crucial role in regulating the biological functions of CRC cells (Lee et al., 2020). Meanwhile, E-cadherin is a well-known epithelial marker, while N-cadherin and Vimentin are regarded as mesenchymal markers. Suppressed EMT is associated with reduced N-Cadherin and Vimentin levels as well as increased E-Cadherin expression (Lin et al., 2020). Furthermore, EMT is a critical mechanism affecting invasion, migration, and metastasis of tumors, also being involved in the occurrence CRC (Spaderna et al., 2006). Therefore, it was reasonable to infer that overexpressed ASXL1 could inhibit EMT in CRC, thus attenuating the progression of CRC. We further confirmed our molecular mechanism underlying LINC00586 in CRC xenograft tissue sections.

**FIGURE 6:**
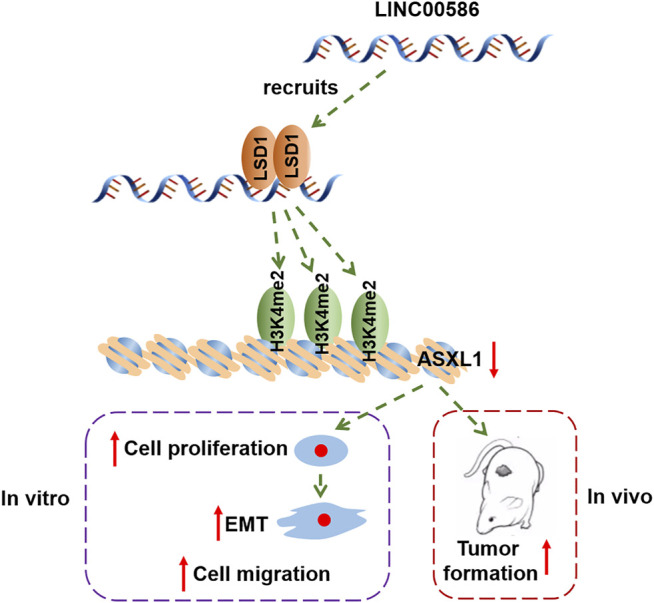
The graphical summary of the mechanism of LINC00586 in regulating CRC progression. LINC00586 epigenetically silenced ASXL1 transcription through LSD1-mediated H3K4me2 demethylation, thereby leading to the development and progression of CRC.

In conclusion, the study provided evidence that LINC00586 knockdown suppressed CRC cell viability, invasion, EMT, and tumorigenicity by mediating ASXL1 expression through LSD1-mediated H3K4me2 demethylation (Figure 6). This study offers a novel insight into the molecular mechanism underlying CRC for theoretical evidence for development of targeted therapies. However, further investigation is still warranted if we want to provide more safe and efficient lncRNA-targeted ways for cancer diagnosis and therapy.

## Data Availability

The original contributions presented in the study are included in the article/Supplementary Material, further inquiries can be directed to the corresponding authors.
